# Clinical characteristics and outcomes in Epstein–Barr virus-positive diffuse large B-cell lymphoma: a multicenter retrospective study

**DOI:** 10.55730/1300-0144.6066

**Published:** 2025-09-02

**Authors:** Ebru KILIÇ GÜNEŞ, Hacer Berna AFACAN ÖZTÜRK, Esra KİRİKTİR, Asena DİKYAR, Bilge UĞUR, Ahmet Kürşad GÜNEŞ, Meltem AYLI

**Affiliations:** 1 Division of Hematology, Department of Internal Medicine, University of Health Sciences, Gülhane Training and Research Hospital, Ankara, Turkiye; 2 Division of Hematology, Department of Internal Medicine, University of Health Sciences, Ankara Etlik City Hospital, Ankara, Turkiye

**Keywords:** Epstein-Barr virus, diffuse large B-cell lymphoma, non-Hodgkin lymphoma, EBV, progression-free survival, overall survival

## Abstract

**Background/aim:**

Epstein–Barr virus-positive (EBV+) diffuse large B-cell lymphoma, not otherwise specified (DLBCL-NOS), is a rare and aggressive subtype of B-cell lymphoma, recognized as a distinct entity in the revised fourth edition of the 2016 World Health Organization (WHO) classification. This retrospective study aims to evaluate the prevalence, clinical and histopathological characteristics, and survival outcomes of patients diagnosed with EBV+ DLBCL-NOS.

**Materials and methods:**

This retrospective study included 92 newly diagnosed DLBCL patients treated at two centers between 2016 and 2024. EBV status was determined by in situ hybridization for EBER, with positivity defined as ≥ 50% nuclear staining in tumor cells.

**Results:**

Among 92 patients, 81 (88.1%) were EBV-negative and 11 (11.9%) were EBV-positive. The median age was 63 years. EBV-positive patients had significantly more advanced-stage disease (100% versus 71.6%, p = 0.049), higher rates of splenic involvement (72.7% versus 38.3%, p = 0.048), elevated LDH levels (median: 773 U/L versus 326 U/L, p = 0.043), and a higher proportion with high-risk IPI scores (IPI ≥ 4: 63.6% versus 27.2%, p = 0.032). No significant differences were observed in age, sex, bulky disease, extranodal involvement, or molecular subtype. Median PFS was 12 months in the EBV-positive group, whereas it was not reached in the EBV-negative group (p = 0.005). EBV positivity was identified as an independent risk factor for inferior PFS (HR: 6.256, 95% CI: 2.398–16.324, p < 0.001), but not for overall survival (OS). Patients with ECOG performance status ≥ 2 and bone marrow involvement also had significantly worse PFS and OS.

**Conclusion:**

EBV+ DLBCL is characterized by distinct clinical features, including advanced-stage disease and poorer PFS. Variability in reported prevalence highlights the need for standardized diagnostic criteria. Further research is essential to develop tailored therapeutic strategies and improve outcomes for this rare lymphoma subtype.

## 1. Introduction

Epstein–Barr virus (EBV) is a ubiquitous human gammaherpesvirus that infects over 90% of the global population [1]. Following primary infection, EBV establishes lifelong latency in B-cells, with periodic reactivation under specific conditions. EBV is implicated in the pathogenesis of various lymphoproliferative disorders and malignancies. These include Hodgkin lymphoma, Burkitt lymphoma, posttransplant lymphoproliferative disorder (PTLD), extranodal NK/T-cell lymphoma, nasal type, and several subtypes of non-Hodgkin lymphoma [2–4]. Beyond lymphoid neoplasms, EBV is also implicated in epithelial tumors such as nasopharyngeal carcinoma and a molecular subtype of gastric carcinoma [5].

EBV-positive diffuse large B-cell lymphoma, not otherwise specified (DLBCL-NOS), is an uncommon and aggressive B-cell lymphoma, recognized as a distinct entity in the 2016 World Health Organization (WHO) classification of lymphoid neoplasms [6]. Originally described by Oyama et al. in 2003, this lymphoma subtype was predominantly observed in elderly patients, who typically had poor prognosis and suboptimal responses to conventional chemotherapy [7]. In 2008, the WHO established a provisional classification for EBV-associated DLBCL in the elderly, defining it as a monoclonal expansion of large B-cells in individuals over 50 years of age without documented immunodeficiency [8]. More recently, both the fifth edition of the WHO Classification of Haematolymphoid Tumours (WHO-5HAEM) [9] and the 2022 International Consensus Classification of Mature Lymphoid Neoplasms (2022-ICC) [10] have provided a similar characterization of EBV+ DLBCL. According to the 2022 ICC, the diagnosis of EBV-positive DLBCL-NOS requires EBER positivity in over 80% of malignant cells, whereas WHO-5HAEM does not specify a strict cutoff and instead emphasizes that the majority of tumor cells should be EBER-positive.

The diagnosis of EBV+ DLBCL-NOS requires histopathological evidence of EBER expression in malignant B-cells, along with the exclusion of other EBV-associated lymphomas such as plasmablastic lymphoma, lymphomatoid granulomatosis, and DLBCL associated with chronic inflammation [11–13]. Despite these criteria, substantial variability remains in the literature regarding cutoff values for EBER positivity, with thresholds ranging from 10% to 90%, contributing to inconsistencies in reported prevalence and prognostic significance [14, 15].

Geographic variation in the incidence of EBV+ DLBCL-NOS has also been reported. In Western countries, prevalence is estimated at 4%–6%, whereas higher rates, ranging from 5% to 28%, have been reported in Asian and Latin American populations [16, 17]. Clinical outcomes are likewise heterogeneous, with some studies suggesting that EBV positivity is associated with worse prognosis, particularly in elderly patients, whereas others have reported no significant impact on survival [18–21].

In this retrospective study, we aim to analyze the prevalence, clinical and histopathological features, and survival outcomes of patients diagnosed with EBV+ DLBCL-NOS.

## 2. Materials and methods

### 2.1. Patients and study design

In this retrospective study, 198 DLBCL patients followed and treated at two centers—Gülhane Training and Research Hospital, Department of Hematology, and Etlik City Hospital, Department of Hematology—between November 2016 and November 2024 were screened. A total of 92 de novo DLBCL patients aged over 18 years, with complete follow-up and treatment data and known EBV status, were included in the study. Patients with double-hit or triple-hit DLBCL, primary central nervous system (CNS) lymphoma, transformation from low-grade lymphoma, primary immunodeficiency or HIV-associated immunodeficiency, secondary malignancies, solid organ transplantation, iatrogenic immunosuppression, or unknown EBV status, as well as those with incomplete diagnostic or treatment data or loss to follow-up, were excluded. Clinical, laboratory, and radiological data were retrospectively reviewed from medical records at initial presentation and during follow-up. Detailed information on disease stage at diagnosis, treatment modalities, relapse dates, and survival durations was recorded.

All patients provided informed consent. The study protocol was conducted in accordance with the ethical principles of the Declaration of Helsinki and aligned with Good Clinical Practice (GCP) standards throughout planning, data collection, and analysis. Ethical approval for this study was obtained from the Institutional Review Board of the Gülhane Faculty of Medicine, University of Health Sciences (decision number 2024–586, 10 December 2024).

### 2.2. Diagnosis, immunohistochemistry, and cell-of-origin classification

The diagnosis of DLBCL was established according to the 2016 WHO classification of lymphoid malignancies [6]. Histopathological evaluation was performed by experienced hematopathologists, confirming morphological findings consistent with DLBCL.

Immunohistochemical evaluation was performed on 4 μm paraffin-embedded tissue sections fixed in formalin and processed using an automated staining system. The following primary antibodies and their respective cut-off values for positivity were used: CD20 (≥ 90% of tumor cells), CD10 (≥ 30% with membranous staining), BCL6 (≥ 30% with nuclear staining), MUM1 (≥ 30% with nuclear staining), BCL2 (≥ 50% with cytoplasmic staining), and Ki-67, with high proliferation defined as ≥ 70%.

The Hans algorithm was used to classify patients into germinal center B-cell-like (GCB) or nongerminal center B-cell-like (non-GCB) subtypes based on immunohistochemistry (IHC) expression of CD10, BCL6, and MUM1 [22].

EBV presence was assessed using in situ hybridization targeting EBER. Tissue sections were processed using an automated hybridization system with commercially available EBER probes. The cut-off value for EBER positivity was determined according to the classification system in use at the time of each patient’s diagnosis. For patients diagnosed before 2022 (38/92), the WHO-2016 classification was applied, which did not specify a strict threshold for EBER positivity in EBV-positive DLBCL. Because of this lack of a standardized cut-off, ≥ 50% EBER positivity was adopted, consistent with previous clinical studies and metaanalyses conducted under WHO-2016 criteria, in which 50% was the most frequently used threshold [14, 15, 18, 20]. For patients diagnosed in or after 2022 (54/92), the 2022 ICC classification was applied, establishing a stricter threshold of ≥ 80% for EBER positivity [9, 10]. Consequently, a cut-off of ≥ 80% was applied for post-2022 diagnoses. As our cohort included patients diagnosed both before and after the 2022 classification update, the EBER cut-off was set at ≥ 50% to ensure consistency across different classification periods.

### 2.3. Clinical assessment indices and treatment

Disease stage was classified for all patients using the revised Ann Arbor staging system [23]. The International Prognostic Index (IPI) was used for prognostic assessment. Treatment response was evaluated according to the revised Lugano criteria, with patients classified as complete response (CR), partial response (PR), stable disease (SD), or progressive disease (PD) [23].

Overall survival (OS) was calculated from the date of initial presentation to death or last follow-up. Progression-free survival (PFS) was defined as the time from diagnosis to disease progression, death, or last follow-up.

Patients were initially treated with either rituximab, cyclophosphamide, doxorubicin, vincristine, and prednisone (R-CHOP) or a lower-intensity regimen (R-mini-CHOP), with treatment chosen by the clinician based on age, performance status, and comorbidities.

### 2.4. Statistical analysis

Categorical data were presented as counts and proportions, whereas continuous variables were reported as medians with ranges. Group comparisons were conducted using the chi-square or Fisher’s exact test for categorical variables, and the Mann–Whitney U test for continuous variables. Kaplan–Meier estimates were used to evaluate PFS and OS, with survival curves compared using the log-rank test. Univariate and multivariate analyses were performed using the Cox proportional hazards regression model to identify independent prognostic predictors. Variables with a p value < 0.10 in univariate analysis were included in the multivariate models. Hazard ratios (HRs) with 95% confidence intervals (CIs) were reported, with statistical significance defined as p < 0.05.

## 3. Results

### 3.1. Baseline characteristics

A total of 92 patients with newly diagnosed DLBCL were included in the study: 81 (88.1%) were EBV-negative and 11 (11.9%) were EBV-positive.

To assess the impact of different EBER cut-off values on the prevalence of EBV-positive DLBCL in our cohort, lower thresholds of 10% and 20% were applied in addition to the predefined 50% cut-off. The analysis showed that reducing the EBER positivity threshold did not change the prevalence of EBV-positive cases in our cohort. The number of EBV-positive patients remained unchanged.

Advanced-stage disease (Stage III–IV) was significantly more frequent in the EBV-positive group compared with the EBV-negative group (100% versus 71.6%, p = 0.049). Similarly, splenic involvement was more frequent in EBV-positive patients (72.7% versus 38.3%, p = 0.048). The median serum lactate dehydrogenase (LDH) concentration was significantly higher in EBV-positive cases than in EBV-negative counterparts (773 U/L versus 326 U/L, p = 0.043). Additionally, a higher proportion of EBV-positive patients were classified as high risk according to the International Prognostic Index (IPI ≥ 4) than EBV-negative patients (63.6% versus 27.2%, p = 0.032).

No significant differences were observed between the two groups in terms of age, sex distribution, bulky disease, bone marrow involvement, extranodal involvement, ECOG performance status, or Ki-67 proliferation index. Likewise, no differences were observed in the cell-of-origin classification (germinal center versus nongerminal center) or in the expression of immunohistochemical markers such as Bcl-2, Bcl-6, CD10, and MUM1.

Table 1 summarizes the baseline demographic, clinical, and pathological characteristics of the patients, along with a comparison of the two groups.

### 3.2. Treatment regimens and response rates

The majority of patients (85.8%) received R-CHOP as first-line therapy, with no significant difference between EBV-positive (90.9%) and EBV-negative (85.2%) groups (p = 0.96). A smaller proportion of patients (14.2%) were treated with the reduced-intensity regimen R-mini-CHOP, with a similar distribution in both groups (p = 0.96).

The median number of cycles administered was six in both EBV-positive and EBV-negative groups (p = 0.847). Treatment completion rates were comparable: 81.8% in EBV-positive and 90.1% in EBV-negative patients (p = 0.843).

The overall response rate (ORR), defined as the sum of CR and PR following first-line treatment, was 82.9% in EBV-positive and 83.6% in EBV-negative patients. CR rates were slightly lower in EBV-positive patients (66.7%) than in EBV-negative patients (76.8%), although the difference was not statistically significant (p = 0.681). Primary refractory disease was observed in 18.2% of EBV-positive and 21% of EBV-negative patients (p = 0.829).

The 6 month mortality rate was higher in EBV-positive patients (18.2%) than in EBV-negative patients (9.9%), although the difference was not statistically significant (p = 0.342). Table 2 summarizes the treatment regimens and response rates for EBV-positive and EBV-negative patients.

### 3.3. Survival rates, univariate and multivariate analysis

#### 3.3.1. Progression-free survival (PFS)

**Figure 1:**
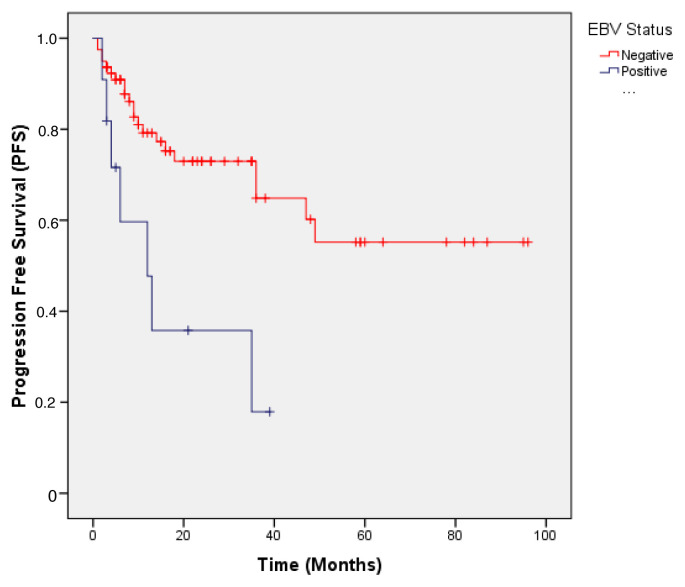
Progression-free survival (PFS) according to EBV status in DLBCL patients.

The median PFS was significantly shorter in EBV-positive than in EBV-negative patients. The median PFS was not reached in the EBV-negative group, whereas it was 12 months in the EBV-positive group (p = 0.005). The hazard ratio (HR) for PFS in EBV-positive patients was 3.536 (95% CI: 1.479–8.456), indicating a significantly higher risk of disease progression. Figure 1 shows Kaplan–Meier curves for progression-free survival according to EBV status.

Univariate analysis revealed that early-stage disease (Stage I–II) was a significant protective factor for PFS (HR: 0.21, 95% CI: 0.05–0.885, p = 0.033). Additionally, IPI ≥ 4 (HR: 2.858, 95% CI: 1.357–6.02, p = 0.006), ECOG performance status ≥ 2 (HR: 4.754, 95% CI: 2.152–10.502, p < 0.001), and bone marrow involvement (HR: 2.342, 95% CI: 1.11–4.943, p = 0.026) were significant predictors of worse PFS. Other variables, including age, sex, extranodal involvement, bulky disease, splenic involvement, and immunohistochemical markers (Bcl-2, Bcl-6, CD10, and MUM1), showed no significant association with PFS.

Multivariate analysis confirmed EBV positivity (HR: 6.256, 95% CI: 2.398–16.324, p < 0.001), ECOG performance status ≥ 2 (HR: 6.489, 95% CI: 2.75–15.307, p < 0.001), and bone marrow involvement (HR: 2.544, 95% CI: 1.181–5.481, p = 0.017) as independent prognostic factors for inferior PFS.

#### 3.3.2. Overall survival (OS)

**Figure 2:**
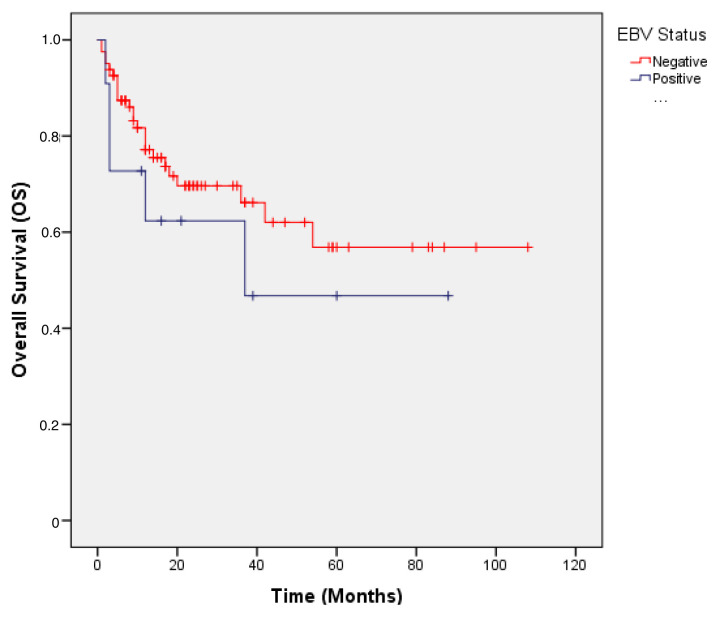
Overall survival according to EBV status in DLBCL patients.

The median OS was not reached in either group during follow-up. The estimated 2 year OS rates were 78.4% for EBV-negative patients and 54.5% for EBV-positive patients, with no statistically significant difference (p = 0.065). Figure 2 shows the Kaplan–Meier survival curves for OS according to EBV status.

Age > 70 years (HR: 2.594, 95% CI: 1.218–5.524, p = 0.018), IPI ≥ 4 (HR: 3.426, 95% CI: 1.641–7.156, p = 0.001), ECOG performance status ≥ 2 (HR: 4.234, 95% CI: 1.946–9.214, p < 0.001), LDH > ULN (HR: 6.696, 95% CI: 1.66–29.496, p = 0.008), and bone marrow involvement (HR: 2.585, 95% CI: 1.243–5.375, p = 0.011) were significantly associated with inferior OS. Factors such as sex, disease stage, extranodal involvement, bulky disease, splenic involvement, and molecular subtype were not significantly associated with OS.

Multivariate analysis identified LDH > ULN (HR: 5.726, 95% CI: 1.349–24.302, p = 0.018) and ECOG performance status ≥ 2 (HR: 3.511, 95% CI: 1.626–7.582, p = 0.001) as independent prognostic factors for poor OS.

## 4. Discussion

In this retrospective study, EBV-positive DLBCL patients had significantly more advanced-stage disease, higher rates of splenic involvement, elevated LDH levels, and increased IPI scores than EBV-negative patients. Furthermore, EBV positivity was identified as an independent risk factor for inferior PFS.

Studies investigating the prevalence of EBV-positive DLBCL have reported variable results, with rates ranging from 5% to 15% [17]. In Western countries, the prevalence is approximately 5%, whereas in Eastern populations it may reach up to 15% [24]. A recent metaanalysis estimated the prevalence of EBV-positive DLBCL at 7.4% (95% CI: 6.2%–10.0%), highlighting significant variability among included studies [15]. In our study, the prevalence of EBV-positive DLBCL was 11.9%.

Variability in prevalence across studies may be due to reliance on small case series and the absence of standardized cut-off values for EBER positivity [17, 25]. A metaanalysis by Gao et al. including 13 studies on EBV-positive DLBCL reported cut-off values for EBER positivity ranging from 10% to 50% [14]. Additionally, no consensus has been reached between the updated WHO-5HAEM (2022) and the 2022-ICC classifications regarding a standardized EBER cut-off value. In a study by Ok et al. including 732 de novo DLBCL cases from Western populations, EBER positivity was observed in 28 cases (4%) when all positive cases were counted. However, prevalence decreased to 3.3% with a 30% cut-off and further declined to 2.2% with a 50% cut-off [24]. In our study, EBER positivity was defined as a cut-off of 50% in patients diagnosed before 2022.

Consistent with previous studies, EBV-positive DLBCL has been associated with older age, male predominance, advanced-stage disease, increased extranodal involvement, B symptoms, elevated LDH levels, and higher IPI scores [14, 17]. Our findings align with these results, as EBV-positive cases in our study presented with advanced-stage disease, elevated LDH levels, and higher IPI scores. However, no significant differences were observed in age, B symptoms, extranodal involvement, or sex distribution between the two groups in our cohort. Additionally, although most EBV-positive cases were classified as the non-GCB subtype by the Hans algorithm, no significant difference in molecular subtypes was observed between the groups. Notably, in contrast to previous studies, our study demonstrated significantly higher rates of splenic involvement in EBV-positive patients.

Among prognostic models, the IPI remains the most widely used for assessing prognosis in DLBCL. However, several studies have reported the limited prognostic value of the IPI in EBV-positive DLBCL, prompting the development of alternative scoring systems [17]. Examples include the Oyama score, which incorporates age and the presence of B symptoms [26], and the GELL score, which evaluates ECOG performance status, extranodal involvement, hypoalbuminemia, elevated LDH, and the platelet-to-lymphocyte ratio [17]. Despite these efforts, novel prognostic models tailored to the unique characteristics of EBV-positive DLBCL remain a critical unmet need.

Response rates to R-CHOP in EBV-positive DLBCL vary considerably, with ORR reported between 50% and 90% and CR rates ranging from 30% to 70%. While some studies have reported lower ORR and CR rates in EBV-positive patients compared with EBV-negative counterparts [27], others have found superior response rates in EBV-positive patients [21]. In our study, ORR did not differ significantly between EBV-positive and EBV-negative groups (82.9% versus 83.6%). Although CR rates were lower in EBV-positive patients (66.7% versus 76.8%), the difference was not statistically significant.

Survival outcomes for EBV-positive DLBCL have varied considerably across studies. While some studies reported no significant difference in OS between EBV-positive and EBV-negative patients receiving chemoimmunotherapy [21, 24, 27], others demonstrated markedly poorer OS in EBV-positive patients [18, 28]. For example, a study by Keane et al., involving 30 EBV-positive patients in Australia, reported shorter OS in the EBV-positive group [29]. Similarly, Bourbon et al. reported significantly reduced OS in EBV-positive patients over 50 years of age in a French cohort [30]. Additionally, a metaanalysis by Gao et al. including 13 studies of EBV-positive DLBCL reported that EBV positivity had a significant negative impact on both PFS and OS [14]. Our findings showed that EBV positivity independently correlated with worse PFS; however, it did not significantly affect OS.

Owing to the frequent expression of CD30 and predominance of the non-GCB molecular subtype in EBV-positive DLBCL, therapies targeting CD30 and CD79b have shown promise in subgroup analyses [31, 32]. However, further clinical studies are needed to confirm their efficacy in this patient population [33].

This study has certain limitations, including the small number of EBV-positive patients, its retrospective design, and the relatively short follow-up duration. Its strengths, however, lie in being based on multicenter real-world data and including both younger and older patients.

In conclusion, EBV-positive DLBCL is a rare but aggressive lymphoma subtype, with most clinical and molecular data derived from small studies and case series, making it challenging to draw definitive conclusions. Although advances in understanding EBV pathobiology have improved insights into this disease, randomized controlled trials are urgently needed to better evaluate outcomes and develop targeted treatment strategies for this unique patient group.

## Figures and Tables

**Table 1:** Baseline demographic, clinical, and pathological characteristics of EBV-positive and EBV-negative DLBCL patients.

		Total Patients	EBV-Negative	EBV-Positive	P value
		**92**	**81 (88.1%)**	**11 (11.9%)**	
**Age**	Median - years	63 (19–84)	63 (19–84)	63 (22–76)	
**Age Groups**	≤70 years	67 (72.8%)	61 (75.3%)	6 (54.5%)	0.146
	>70 years	65 (27.2%)	20 (24.7%)	5 (45.5%)	
**Sex**	Male	47 (51.1%)	42 (51.9%)	5 (45.5%)	0.690
	Female	45 (48.9%)	39 (48.1%)	6 (54.5%)	
**Stage**	Early Stage (I–II)	23 (25%)	23 (28.4%)	-	**0.049**
	Advanced Stage (III–IV)	69 (75%)	58 (71.6%)	11 (100%)	
**LDH**	Median U/L	357 (134–2155)	326 (134–2155)	773 (170–1510)	**0.043**
**LDH>ULN**	Yes	67 (72.8%)	59 (72.8%)	8 (72.7%)	1.000
**Bcl-2**	Positive	69 (75%)	61 (75.3%)	8 (72.7%)	0.853
**Bcl-6**	Positive	76 (82.6%)	67 (82.7%)	9 (81.8%)	0.941
**CD10**	Positive	29 (31.5%)	27 (33.3%)	2 (18.2%)	0.492
**MUM-1**	Positive	58 (63%)	49 (60.5%)	9 (81.8%)	0.203
Molecular Subtype*	Germinal Center	33 (35.9%)	28 (34.6%)	3 (27.3%)	0.745
	Nongerminal Center	59 (64.1%)	53 (65.4%)	8 (82.7%)	
**Ki67 Proliferation Index**	>70%	32 (34.8%)	29 (35.8%)	3 (27.3%)	0.742
	≤70%	60 (65.2%)	52 (64.2%)	8 (72.7%)	
**ECOG Performance Status**	0–1	60 (65.2%)	53 (65.4%)	7 (63.7%)	-
	2	22 (23.9%)	20 (24.8%)	2 (18.2%)	
	3	10 (10.9%)	8 (9.8%)	2 (18.2%)	
**ECOG ≥2**	Yes	32 (44.8%)	28 (34.6%)	4 (36.4%)	1.000
**Extranodal Involvement**	Yes	45 (48.9%)	38 (46.9%)	7 (63.6%)	0.349
**Splenic Involvement**	Yes	39 (42.4%)	31 (38.3%)	8 (72.7%)	**0.048**
**Bulky Disease**	Yes	20 (21.7%)	17 (21%)	3 (27.3%)	0.699
**Bone Marrow Involvement**	Yes	27 (29.3%)	24 (29.6%)	3 (27.3%)	0.941
**IPI Risk Group**	Low	17 (18.5%)	17 (21%)	-	-
	Low-Intermediate	22 (24.1%)	20 (24.7%)	2 (18.2%)	
	High-Intermediate	24 (25.9%)	22 (27.2%)	2 (18.2%)	
	High	29 (31.5%)	22 (27.2%)	7 (63.6%)	
**IPI High Risk ( ≥4)**	Yes	29 (31.5%)	22 (27.2%)	7 (63.6%)	**0.032**

**Table 2:** Treatment regimens, response rates, and outcomes in EBV-positive and EBV-negative DLBCL patients.

		Total Patients	EBV-Negative	EBV-Positive	p value
		**92**	**81 (88.1%)**	**11 (11.9%)**	
**Treatment Regimens**	R-CHOP	79 (85.8%)	69 (85.2%)	10 (90.9%)	0.960
	R-mini-CHOP	13 (14.2%)	12 (14.8%)	1 (9.1%)	
**Total Cycles Received**	Median	6	6 (1–6)	6 (2–6)	0.847
**Completed First Line Treatment**	Yes	82 (89.1%)	73 (90.1%)	9 (81.8%)	0.843
**Mortality within first 6 months**	Yes	10 (10.9%)	8 (9.9%)	2 (18.2%)	0.342
**Response to First Line treatment**	CR	62 (75.6%)	56 (76.8%)	6 (66.7%)	-
	PR	6 (7.3%)	5 (6.8%)	1 (11.1%)	
	SD	2 (2.5%)	2 (2.7%)	-	
	PD	12 (14.6%)	10 (13.7%)	2 (22.2%)	
**Response to First Line Treatment**	CR	62 (75.6%)	56 (%76.8)	6 (66.7%)	0.681
	Non-CR	20 (24.4%)	17 (23.2%)	3 (33.4%)	
**Primary Refractory Disease**	Yes	19 (20.7%)	17 (21%)	2 (18.2%)	0.829
